# Focus on leveling the hidden: managing impacted maxillary canines

**DOI:** 10.1590/2177-6709.29.5.e24spe5

**Published:** 2024-10-07

**Authors:** Orlando Motohiro TANAKA, André WEISSHEIMER, Matheus Melo PITHON, Gil Guilherme GASPARELLO, Eustáquio Afonso ARAÚJO

**Affiliations:** 1 Pontifícia Universidade Católica do Paraná, PPGO-PUCPR (Curitiba/PR, Brazil).; 2 Harvard University, School of Dentistry, Department of Orthodontics (Boston, USA).; 3 State University of Southwest Bahia, School of Dentistry, Department of Orthodontics (Jequié/BA, Brazil).; 4 Saint Louis University, Center for Advanced Dental Education (Saint Louis, USA).

**Keywords:** Impacted maxillary canine, Diagnosis, Biomechanics, Canino superior impactado, Diagnóstico, Biomecânica

## Abstract

**Introduction:**

The long pathway that the canines take as they emerge into the maxillary arch makes them vulnerable to disruption during their natural emergence time. The process of planning treatment for impacted maxillary canine (IMC) presents significant challenges, underscoring the need for careful consideration and expertise.

**Objective:**

The aim of this article was to shed light on these complexities by discussing clinical case studies involving IMC, providing insights into the intricacies of their management.

**Conclusions:**

The management of IMC within orthodontics presents a multifaceted challenge that include the necessity for precise diagnostic processes, prudent use of cone beam computed tomography (CBCT), the strategic selection between open and closed exposure techniques, a in-depth understanding of the specific orthodontic biomechanics involved, and a keen awareness of potential adverse outcomes such as ankylosis, prolonged treatment times, root resorption, and additional complications.

## RESUMO

**Introdução:**

O longo caminho que os caninos percorrem até irromper na arcada superior os torna vulneráveis a interrupções durante a erupção fisiológica. A etapa de planejamento do tratamento para caninos superiores impactados (CSI) apresenta desafios significativos, destacando a necessidade de consideração cuidadosa e expertise.

**Objetivo:**

O objetivo do presente artigo é esclarecer esses aspectos, discutindo relatos de casos clínicos envolvendo CSI, fornecendo percepções sobre a complexidade de seu manejo.

**Conclusões:**

O manejo do CSI na Ortodontia apresenta um desafio multifacetado, que inclui a necessidade de um diagnóstico preciso, o uso prudente de tomografia computadorizada de feixe cônico (TCFC), a seleção estratégica entre as técnicas de exposição aberta ou fechada, uma compreensão aprofundada da biomecânica ortodôntica específica envolvida, e uma consciência ampla dos possíveis resultados adversos, como anquilose, tempo de tratamento prolongado, reabsorção radicular e complicações adicionais.

## INTRODUCTION

Tooth impaction is defined as a condition in which the tooth remains embedded in the oral mucosa or intraosseous structures after its natural eruption.^1^ When a permanent tooth fails to erupt within one year after its physiological period, it is considered impacted.^2^

The long pathway that the canines take as they emerge into the maxillary arch makes them vulnerable to disruption during their natural emergence time, at the age of 11-12 years,^3^ and after the third molars, canines are the most frequently impacted teeth, with a frequency of 1-3%.^4^ Early detection and intervention, along with coordinated surgical and orthodontic treatments, can guide impacted maxillary canines (IMC) to a proper position in the dental arch.^5^

The traditional approach to orthodontic-surgical management of IMC typically involves the surgical exposure of the tooth, utilizing either the open exposure^6^ or closed exposure^7^ techniques, followed by the intraoperative bonding of an orthodontic button linked to a chain, which is then attached to the archwire of the fixed appliance. However, the actual procedure is much more complex.

The process of planning treatment for IMC presents significant challenges, underscoring the need for careful consideration and expertise. Thus, the aim of the present article is to throw light on these complexities, by discussing clinical cases involving IMC, providing insights into the intricacies of their management.

## DIAGNOSIS OF IMPACTED CANINES

**Figure 1:**
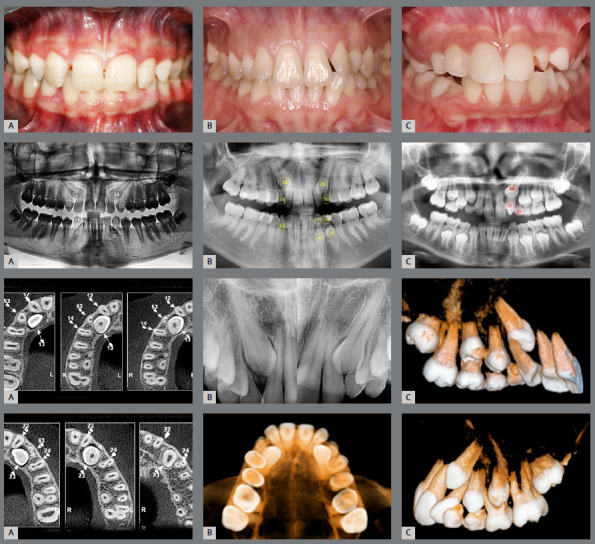
Frontal clinical photographs, panoramic and periapical radiographs, and CBCT for the diagnosis of the position of the impacted maxillary canine. A) Distal inclination of the laterals, and the canines positioned palatally. B) Distal inclination of the laterals, and canines without deviation. C) Distal inclination of the left lateral, and the canine positioned labially.

Localization of IMC position is essential for accurate orthodontic treatment as well as prognosis. The first step in managing IMC is accurate diagnosis, to determine the position and path of eruption of the canine, its effect on adjacent teeth, and the overall dental health of the patient. This involves clinical (Fig 1) and imaging examination. 

Clinical signs of IMC include delayed eruption of the canine, retention of the deciduous canine, absence of the labial bulge, presence of a palatal bulge, and tipping of the adjacent incisor.^5^ The absence of a canine bulge by age 11 might not always mean impaction. Palpating the alveolar process near the lateral incisor can help assess the canine’s position before it emerges. A missing labial bulge in 9- or 10-year-olds should raise concerns, leading to radiographic evaluations for a definitive diagnosis.^8^

The use of cone beam computed tomography (CBCT) (Fig 1) enables a more precise assessment of the exact position, and the intimate relation between the canine crown and the neighboring teeth is of special interest during orthodontic treatment planning for IMC, minimizing potential complications.^9^ In situations in which CBCT is not available at the dental practice, panoramic radiography (Fig 1) can aid in the localization of IMCs.^10^

## OPEN AND CLOSED EXPOSITION TECHNIQUES

**Figure 2:**
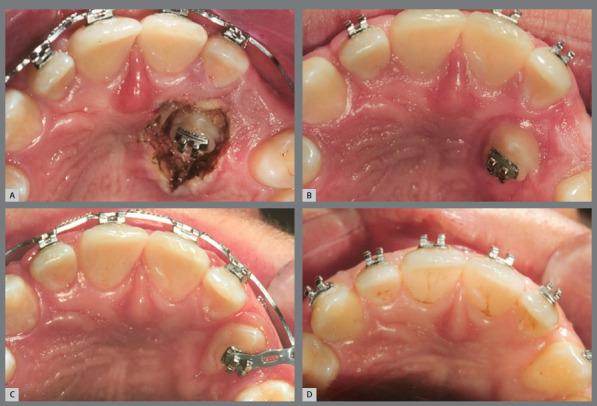
Open exposure of palatally impacted maxillary canine.

The conventional method of orthodontic-surgical treatment of IMC consists in surgical tooth exposure, using the open exposure, a surgical procedure to reveal the tooth (Fig 2), followed by the direct bonding of an orthodontic attachment, which is connected to the arch of the fixed appliance or to the aligners. When the canine is located near the surface, the open exposure method is preferred.^6^

**Figure 3:**
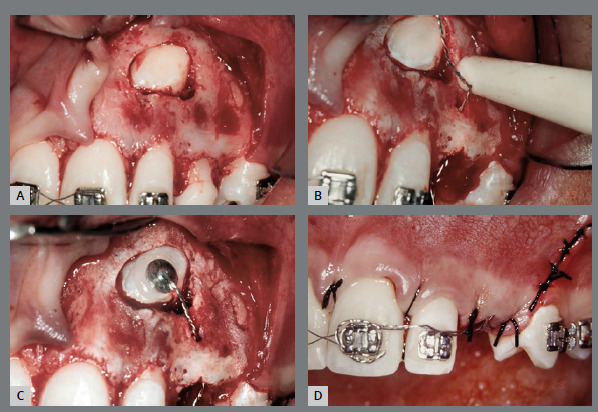
After attaching the hook, the wire is positioned to direct the tooth to its correct position in the occlusion line.

**Figure 4:**
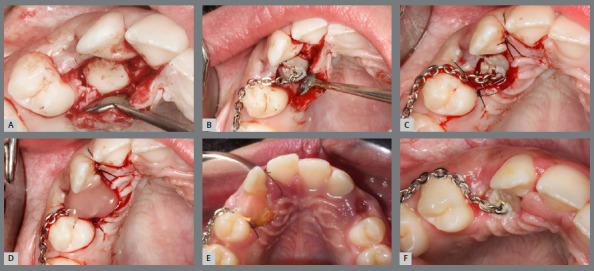
Closed exposure of palatally impacted maxillary canine, with a light-cure periodontal surgical dressing.

Conversely, closed exposure is employed to guide the movement of the tooth within the bone and gingival tissues without immediate exposure (Figs 3 and 4). For canines situated deeper within the bone, the closed exposure approach is more suitable.^7^

**Figure 5:**
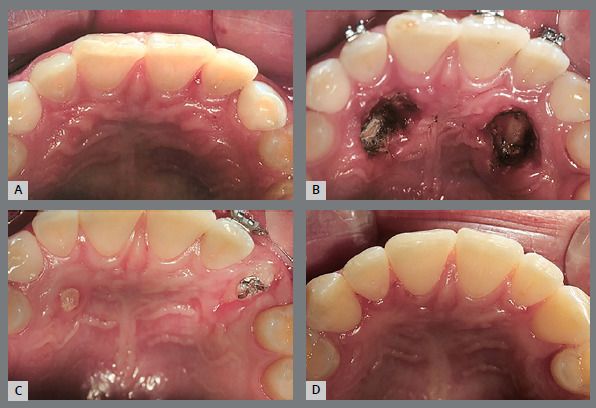
Open exposure, whereas it should be a closed exposure of palatally impacted maxillary canine.

An inadequate exposure technique due to a lack of understanding or communication with the professional performing the exposure can lead to outcomes that complicate the traction phase. In Figure 5, both canines were positioned too deep, and neither a chain nor a hook was used. For very deep cases, creating a perforation in the crown is a reliable method to prevent the need for a second surgical procedure. 

**Figure 6:**
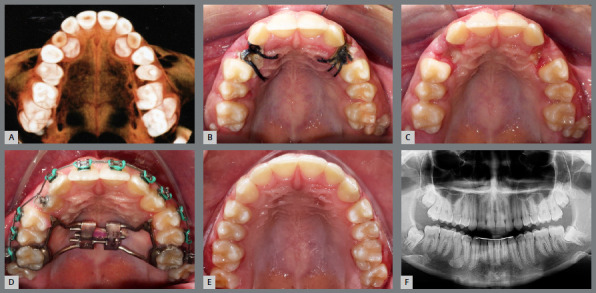
Extraction of the deciduous canines, and exposure of the permanent canines. It could have been an open exposure, and no sutures would be necessary.

**Figure 7:**
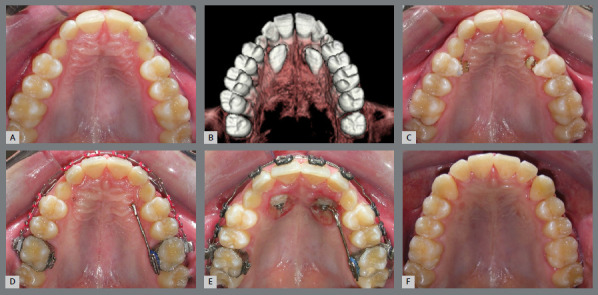
Closed exposure, whereas it should be used an open exposure of palatally impacted maxillary canine.

In Figure 6, the canines were positioned close to the level of the gingiva, eliminating the need for sutures or closed exposure. In Figure 7, the stainless steel wire was excessively thin and fractured during the initial traction process, requiring a second exposition.

There is some evidence suggesting that there are no significant differences in outcomes between open and closed surgical exposures for IMC; however, the quality of this evidence is considered low.^11^ Additionally, the closed exposure technique is associated with a higher incidence of ankylosis, which occurs in 14.5% of cases, as opposed to 3.5% in the open exposure technique.

Orthodontists commonly refer patients to oral surgeons or periodontists for the exposure and alignment of impacted teeth. Although these procedures might also be performed by other specialists, orthodontists have a legal responsibility for obtaining informed consent. It is recommended that orthodontists use an informed consent form to maintain ethical and legal diligence in such cases.^12^

## TYPE OF ORTHODONTIC BIOMECHANICS

The traction of palatally IMC is one of the greatest challenges of orthodontic treatment, and success depends on many factors: incisor overlap, vertical height, angulation, and apex position. 

In patients with IMC, no dental crossbite, and lack of space in the maxillary arch, maxillary expansion^13^ results in significant improvement of the sector in which the canine is positioned, and decreases the need for major orthodontic intervention in the long term, compared to extraction of deciduous canine or a wait-and-see attitude.^14^

**Figure 8:**
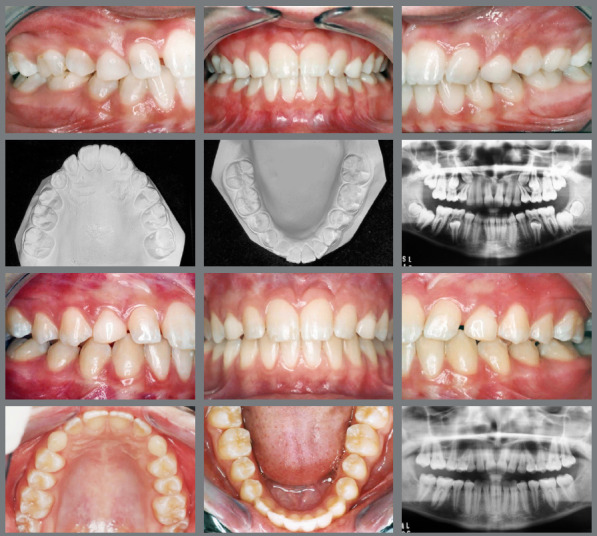
Both impacted maxillary canines were extracted and the deciduous were maintained.

**Figure 9:**
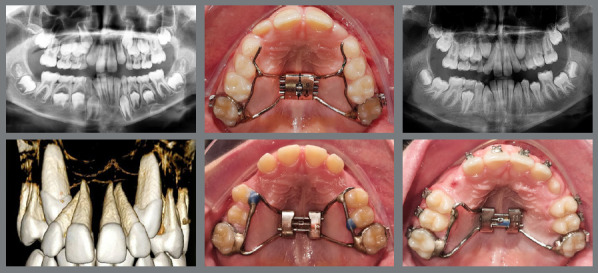
The rapid maxillary expansion facilitated the eruption of both maxillary canines.

**Figure 10:**
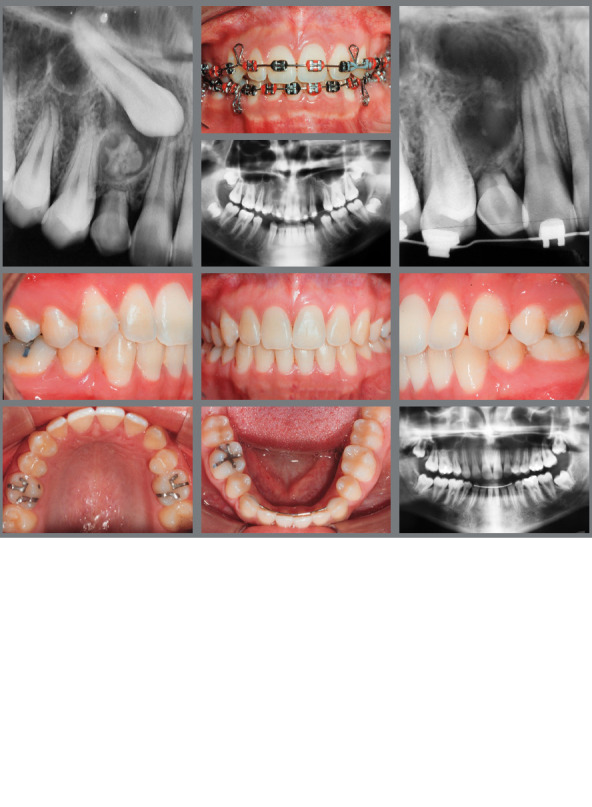
Three first premolars and the right impacted maxillary canine were extracted, and the space was closed by retracting the incisors, to improve the facial profile.

**Figure 11:**
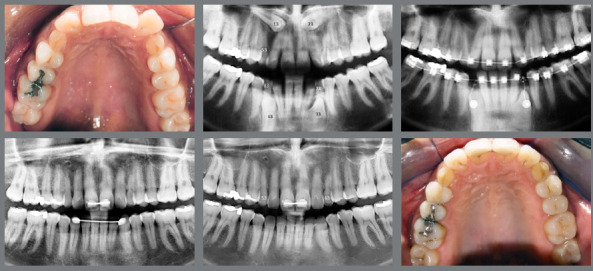
In an adult patient with all four canines impacted, both mandibular canines were brought into alignment, while both impacted maxillary canines were extracted, and the deciduous canines remained in place for the 12-year follow-up.

Essential factors in the biomechanical strategies used in orthodontic movement of IMCs - such as the direction of applied forces, which may be light and continuous, the nature of anchorage, and the sequence of orthodontic movements - are fundamental to successful outcomes. The specific biomechanical approach can vary significantly, being individualized to the unique requirements of each case. There is a variety of orthodontic interventions for managing IMCs, ranging from non-intervention^15^ (Fig 8), interceptive treatments^16^ (Fig 9), extraction^17^ (Fig 10), maintenance of the deciduous canine^18^ (Fig 11), and autotransplantation.^19^

**Figure 12:**
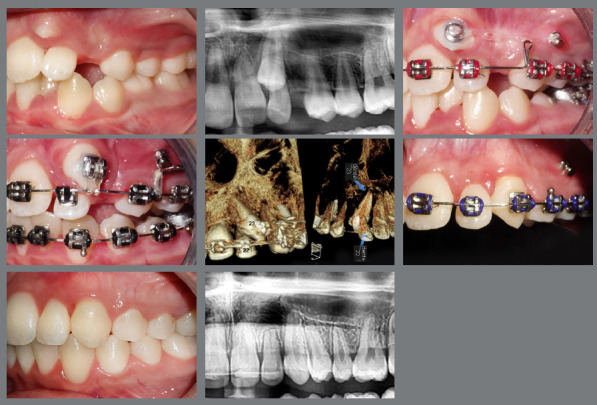
A TAD was used to achieve distal inclination of the left maxillary canine. Significant torque was applied to the lateral incisor, which might have resulted in mild root resorption.

**Figure 13:**
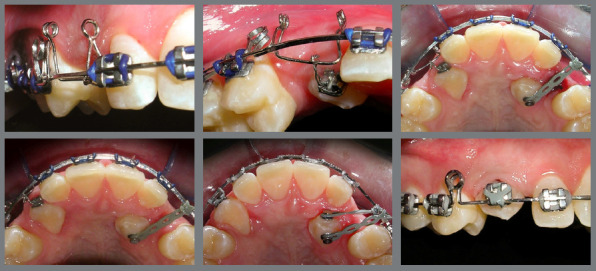
The 0.016-in stainless steel multiloop wire enables the impacted maxillary canine to move labially without the need for a bite block.

For palatally IMC, the recommended approach involves initially tractioning the tooth downward, followed by moving it toward the labial side. The multiloop arch with double helix is efficient for moving the canine to the occlusion line when it is positioned palatally and in crossbite, eliminating the necessity of a bite block^20^ (Fig. 13).

In contrast, for canines positioned labially, whether facing mesially or distally, the first step is to align the tooth within the dental arch, followed by vertical traction, to position it correctly in occlusion. 

When only a mesial or distal inclination correction is needed in the first phase, the use of temporary anchorage devices (TAD) provides more effective biomechanical control, and is particularly effective, as shown in Figure 12.

## UNCERTAINTIES IN TREATMENT

There are inherent uncertainties in treating IMC, such as variations in response to orthodontic forces, potential relapse, and patient-specific factors, like age and oral hygiene. These uncertainties demand a flexible and adaptive treatment approach, often individualized to the individual patient’s needs. 

Ankylosis is a potential complication in treating impacted canines. The risk increases with the age of the patient and the duration of impaction, the anatomic position of the canine’s root apex and its adjacent anatomic structures. 

**Figure 14:**
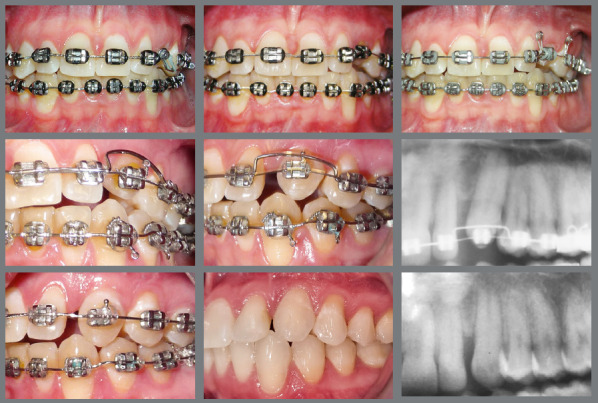
Intrusion of the adjacent teeth during leveling. The apicotomy was a contributing factor to the successful alignment of the teeth to the occlusion line.

The apicotomy surgical technique can be an effective option as an adjunctive treatment for ankylosed or dilacerated IMC after the failure of conservative orthodontics biomechanics^21^ ^,^ ^22^ (Fig 14).

The duration of the surgical/orthodontic treatment, ranging from 18 to 30 months,^23^ is a particularly troubling clinical problem, because it prolongs the orthodontic treatment duration.^24^ This is related to many factors, including: the patients’ age, the severity of dental crowding, the initial inclination of the IMC, the buccolingual IMC’s position, the distance from the occlusal plan and the periodontal health.^25^ The patient should be informed of the possibility of failure, a factor that, together with the increased treatment time, must be brought into the decision-making process from the outset.^26^

It is essential that patients -or their legal guardians, in the case of minors- are comprehensively briefed about the proposed treatment strategies, the associated risks, and the viable alternatives.^12^

**Figure 15:**
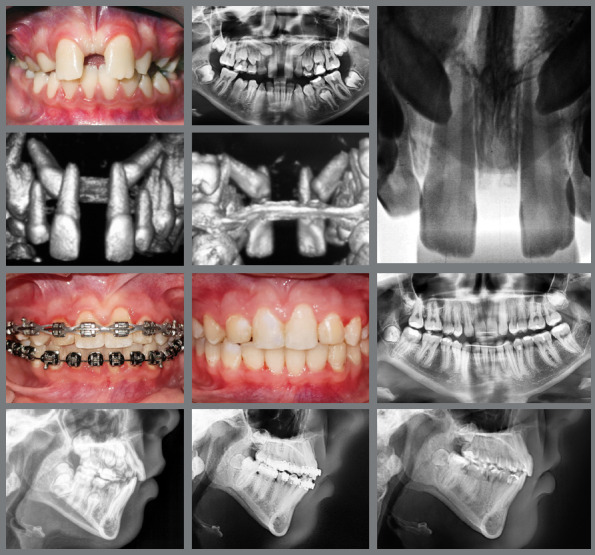
The maxillary canines in the positions of the lateral incisors. The use of Class III intermaxillary elastics favored the loss of maxillary anchorage and the maintenance of the mandibular incisors on the bone base. However, poor oral hygiene led to the formation of white spots.

Patient adherence to treatment protocols, particularly in the use of orthodontic devices such as elastics, is essential for achieving successful results. Moreover, maintaining optimal oral hygiene is critical to avoid periodontal issues and cavities, which can pose challenges due to the brackets and wires used in orthodontic treatments.^12^ Non-compliance by the patient can hinder the quality of the final outcome, as illustrated in Figure 15.

**Figure 16:**
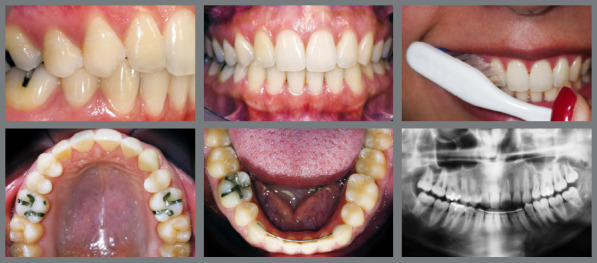
Gingival recession observed after a 12-year follow-up period.

After replacing the maxillary canine with the reshaped first premolar, occlusal balance must be established, and the lateral guidance on the canine should be well-dimensioned.^27^ Figure 16 illustrates a gingival recession with cervical abrasion on the maxillary right premolar associated with improper brushing technique.

## INTERDISCIPLINARY APPROACH

**Figure 17:**
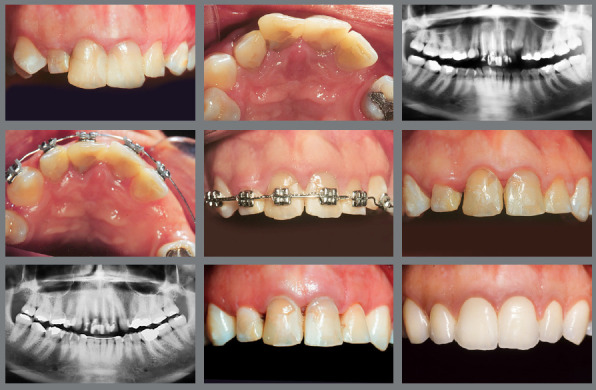
A multidisciplinary approach involving orthodontics for alignment, surgical intervention for tooth exposure, periodontics for crown lengthening, and esthetic dentistry for the final restoration.

Managing IMC often demands a multidisciplinary approach, involving collaboration among orthodontists, oral surgeons, and, in some instances, periodontists. This teamwork is important for complex cases in which surgical exposure, orthodontic alignment, and periodontal health must be concurrently managed to attain optimal aesthetic outcomes (Fig 17).

## POST-TREATMENT MONITORING AND RETENTION

After achieving successful alignment of an IMC, implementing long-term retention strategies is necessary to preserve tooth positioning and reduce relapse chances. The use of fixed retainers, when appropriate, plays a significant role in maintaining alignment. Additionally, scheduling routine follow-up visits is essential for evaluating treatment stability and efficiently managing any arising complications, thereby ensuring the effectiveness of the orthodontic treatment.

## DISCUSSION

In the treatment of IMC, early detection and preventive strategies are important. When tooth impaction occurs, a coordinated approach involving orthodontic treatment and surgical exposure is recommended for proper alignment, necessitating a synergistic effort between orthodontists and oral surgeons.^5^ IMC often is undiagnosed, due to the seemingly correct alignment of anterior teeth, leading to delayed orthodontic referrals until the eruption of second molars. 

The timing of intervention and the patient’s age are fundamental in influencing the success of treatment. Intervention during adolescence is advantageous due to the lower bone density and better orthodontic treatment response. The American Association of Orthodontists recommends early orthodontic evaluations to detect any dental problems, ideally by the age of 7, when enough permanent teeth are present for an assessment. Radiographic examinations are critical for early detection and to avoid complications such as root resorption.^28^

There are various methods to treat an ankylosed IMC, with luxation being the most common, despite its low success rate and associated risks. Surgical repositioning is an alternative, though it is less favored due to high morbidity.^29^ The last attempt is the apicotomy.^21^ The complexity and duration of orthodontic treatment for severe IMC underscore the importance of early detection to employ interceptive methods, which can prompt spontaneous correction and normal eruption of the IMC.

The management of IMC often involves a prolonged treatment duration, emphasizing the need for diligent monitoring to ensure the health of the tooth and surrounding structures. The main complication arising from IMCs is adjacent teeth’s root resorption, which tends to stabilize long-term after correctly positioning the canine.^30^

While orthodontic treatment may have side effects such as mild discomfort and gingival recession, the advantages of successfully aligning an IMC, such as improved functionality, aesthetics, and prevention of further dental issues, are significant. 

## FINAL CONSIDERATIONS

Managing impacted maxillary canines (IMC) in orthodontics is complex, and requires accurate diagnosis, careful use of CBCT, and choosing between open and closed exposure methods. Understanding orthodontic biomechanics and being aware of risks like ankylosis and root resorption are crucial. Success also depends on teamwork across disciplines, considering patient-specific factors, and clear communication about possible side effects. Coordination with the professional performing the canine exposure is essential.
